# LKB1 in Intestinal Epithelial Cells Regulates Bile Acid Metabolism by Modulating FGF15/19 Production

**DOI:** 10.1016/j.jcmgh.2021.12.017

**Published:** 2021-12-29

**Authors:** Yeji Kim, Sohyeon Lee, Seungil Kim, Tae-Young Kim, Su-Hyun Lee, Jae-Hoon Chang, Mi-Na Kweon

**Affiliations:** 1 Mucosal Immunology Laboratory, Department of Convergence Medicine, Asan Medical Center, University of Ulsan College of Medicine, Seoul, Republic of Korea; 2 Digestive Diseases Research Center, University of Ulsan College of Medicine, Seoul, Republic of Korea; 3 College of Pharmacy, Yeungnam University, Gyeongsan, Republic of Korea

**Keywords:** Bile Salt Hydrolase, IEC, LKB1, Retinoic Acid, T-βMCA, 9cRA, 9-cis retinoic acid, AMPK, AMP-activated protein kinase, atRA, all-trans retinoic acid, BA, bile acid, BSH, bile salt hydrolase, CA, cholic acid, CCK, cholecystokinin, CYP7a1, cholesterol 7α-hydroxylase, DCA, deoxycholic acid, ELISA, enzyme-linked immunoabsorbant assay, FGF15/19, fibroblast growth factor 15/19, FXR, farnesoid X receptor, GSEA, Gene Set Enrichment Analysis, HFD, high-fat diet, IEC, intestinal epithelial cell, ISC, intestinal stem cell, LKB1, liver kinase B1, OCA, obeticholic acid, PBS, phosphate-buffered saline, PCR, polymerase chain reaction, qPCR, quantitative polymerase chain reaction, RA, retinoic acid, RXR, retinoid X receptor, SI, small intestine, T-βMCA, tauro-β-muricholic acid, TCA, taurocholic acid

## Abstract

**Background & Aims:**

Liver kinase B1 (LKB1) is a master upstream protein kinase involved in nutrient sensing and glucose and lipid metabolism in many tissues; however, its metabolic role in intestinal epithelial cells (IEC) remains unclear. In this study, we investigated the regulatory role of LKB1 on bile acid (BA) homeostasis.

**Methods:**

We generated mice with IEC-specific deletion of LKB1 (LKB1^ΔIEC^) and analyzed the characteristics of IEC development and BA level. In vitro assays with small interfering RNA, liquid chromatography/mass spectrometry, metagenomics, and RNA-sequencing were used to elucidate the regulatory mechanisms underlying perturbed BA homeostasis.

**Results:**

LKB1 deletion resulted in abnormal differentiation of secretory cell lineages. Unexpectedly, BA pool size increased substantially in LKB1^ΔIEC^ mice. A significant reduction of the farnesoid X receptor (FXR) target genes, including fibroblast growth factor 15/19 (FGF15/19), known to inhibit BA synthesis, was found in the small intestine (SI) ileum of LKB1^ΔIEC^ mice. We observed that LKB1 depletion reduced FGF15/19 protein level in human IECs in vitro. Additionally, a lower abundance of bile salt hydrolase-producing bacteria and elevated levels of FXR antagonist (ie, T-βMCA) were observed in the SI of LKB1^ΔIEC^ mice. Moreover, LKB1^ΔIEC^ mice showed impaired conversion of retinol to retinoic acids in the SI ileum. Subsequently, vitamin A treatment failed to induce FGF15 production. Thus, LKB1^ΔIEC^ mice fed with a high-fat diet showed improved glucose tolerance and increased energy expenditure.

**Conclusions:**

LKB1 in IECs manages BA homeostasis by controlling FGF15/19 production.

## Graphical abstract

**Figure:**
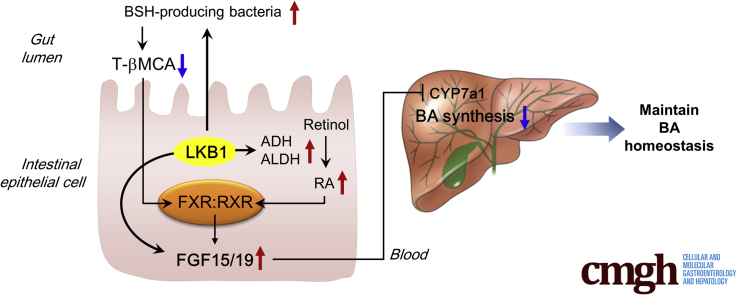

**Summary**

We observed the regulatory role of intestinal liver kinase B1 (LKB1) in bile acid synthesis through fibroblast growth factor (FGF) 15 signaling. LKB1 depletion directly reduced FGF15/19 secretion in an farnesoid X receptor-independent manner. It also inhibited FGF15/19 production through increased Tauro-β-muricholic acid, decreased bile salt hydrolase-producing bacteria, and impaired retinol metabolism.

Liver kinase B1 (LKB1), also known as serine-threonine kinase 11, is an upstream regulator of AMP-activated protein kinase (AMPK) and 12 structurally related kinases.^1^ LKB1 is important in regulating glucose and lipid metabolism in various metabolic tissues, including hepatic LKB1-suppressed gluconeogenesis and inhibition of amino acid catabolism.^2^, ^3^ Previous research has shown that adipocyte-specific deletion of LKB1 promotes brown adipose tissue mass and consequently prevents high-fat diet (HFD)-induced obesity.^4^ In addition, LKB1 in the skeletal muscle controlled fatty acid oxidation, whole-body insulin sensitivity, and glucose homeostasis.^5^, ^6^, ^7^ The small intestine (SI) is the site of nutrient absorption and transport,^8^ gut hormone secretion,^9^ and of diverse microbiota that influence energy, glucose, and lipid homeostasis.^10^ Previous reports have shown that intestinal epithelial cell (IEC)-specific deletion of LKB1 induced altered development of Paneth and goblet cell lineages in the SI, and reduced interleukin-18 and antimicrobial peptide production in the colon.^11^^,^^12^ Nevertheless, the exact role of intestinal epithelial LKB1 in metabolism remained uninvestigated.

Bile acid (BA) synthesized in the liver is secreted into the SI to facilitate digestion and absorption of dietary lipids.^13^ Primary BA is produced from cholesterol in the liver by cholesterol 7α-hydroxylase (CYP7a1), and then conjugated to glycine and taurine.^14^ After transit through the SI to perform their function, approximately 95% of BA is then reabsorbed from the ileum and returned to the liver.^15^ The size of the BA pool, defined as total BA levels in enterohepatic circulation within the liver, intestine, and gallbladder, is tightly regulated by a feedback mechanism that allows the liver to control the synthesis in response to BA levels.^15^^,^^16^ Dysregulation of BA homeostasis affects lipid and glucose metabolism, and consequently contributes to obesity, fatty liver diseases, and type 2 diabetes.^15^^,^^17^^,^^18^

BA receptor expression in the gut and BA metabolism by gut microbiota are essential for maintaining gut homeostasis. The farnesoid X receptor (FXR), a nuclear BA receptor in hepatocytes and enterocytes, is a key regulator of BA synthesis.^16^^,^^19^ Hepatic FXR activated by BA suppresses CYP7a1 expression.^20^ Activated FXR in the SI ileum also inhibits BA synthesis through the secretion of fibroblast growth factor 15/19 (FGF15 and 19 in mice and humans, respectively), which reaches the liver and activates FGF receptor 4 on hepatocytes, resulting in the repression of CYP7a1 expression.^21^ Although some BA acts as agonists for FXR, some may exhibit antagonist effects. As gut microbiota metabolizes BA, including deconjugation of glycine or taurine by bile salt hydrolase (BSH) and transformation into secondary BA by 7α-dehydroxylase,^22^ they can regulate BA synthesis by controlling the balance between FXR activator and inhibitor.^23^ For example, BSH-producing bacteria can regulate BA synthesis by reducing the levels of tauro-β-muricholic acid (T-βMCA), known as an FXR antagonist.^24^^,^^25^ Moreover, metformin ameliorates metabolic disorders in part through inhibiting intestinal FXR signaling by reduction the proportion of BSH-producing bacteria, increasing conjugated BA, and acting as an FXR antagonist.^26^ Thus, intestinal FXR-FGF15/19 axis regulation has been suggested as a potential therapeutic target for obesity, liver, and metabolic diseases.^27^^,^^28^

Here, we show that LKB1^ΔIEC^ mice had an increased BA pool size, decreased SI ileal FGF15/19, and increased hepatic CYP7a1 expression. We also observed elevated levels of the FXR antagonist (ie, T-βMCA) with a reduction in BSH-producing bacteria in LKB1^ΔIEC^ mice. Low expression of FGF15/19 was also associated with impaired retinol metabolism in these mice. Accordingly, we observed improved glucose tolerance and increased energy expenditure in HFD-fed LKB1^ΔIEC^ mice.

## Results

### Intestinal Epithelial LKB1 Deletion Affects Intestinal Stem Cell Proliferation and Secretory Lineage Differentiation

**Figure 1:**
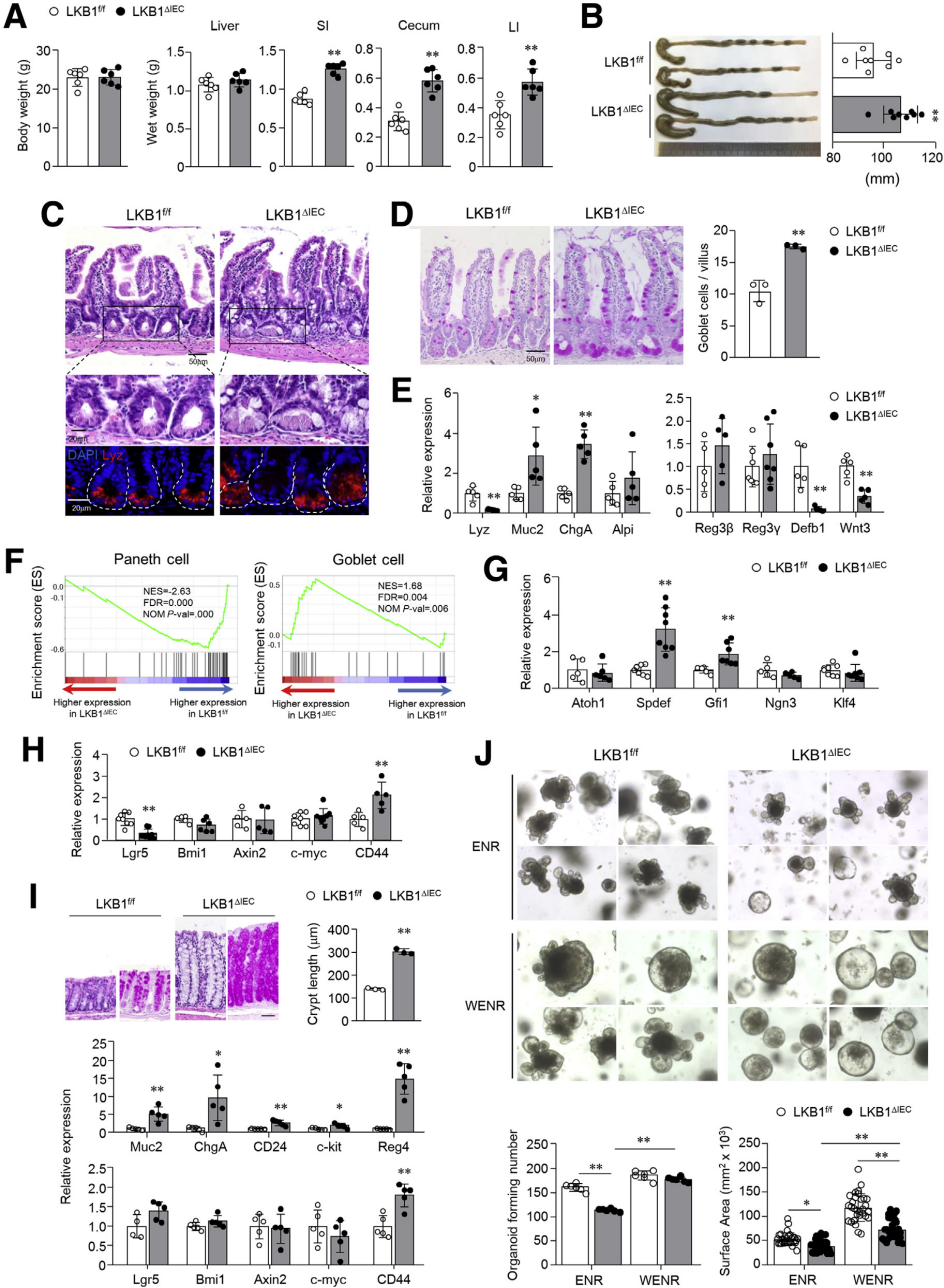
**Secretory lineage cells were abnormally differentiated in the small intestine of LKB1**^**ΔIEC**^**mice.** (*A*) Weights of the body, liver, SI, cecum, and LI of LKB1^f/f^ and LKB1^ΔIEC^ mice at 12 weeks old (n = 6/group). (*B*) Colon lengths of mice (n = 8/group). (*C*) LKB1^ΔIEC^ mice have an abnormal morphology of the SI. Representative images of H&E and immunofluorescence staining for lysozyme (*red*). Bar = 50 μm (H&E) and 20 μm (immunofluorescence). (*D*) Representative images of PAS staining and quantification of PAS-positive goblet cells per villus in SI ileum were shown (n = 3/group). Bar = 50 μm. (*E*) Gene expression for IECs and Paneth cell secretomes in the SI ileum were examined by qPCR (n = 5/group). (*F*) GSEA of RNA-seq data in the SI ileum (n = 3/group). mRNA levels of transcription factors for secretory lineage (*G*), markers for intestinal stem cells, and Wnt target genes (*H*) were assessed by qPCR (n = 5–8/group). (*I*) Representative images of the colon stained with H&E and PAS, and quantification of crypt depth in the distal colon of LKB1^f/f^ and LKB1^ΔIEC^ mice (n = 3/group). mRNA levels of IECs in the colon were evaluated by qPCR (n = 4–5/group). (*J*) Representative images of organoids and quantification of organoid-forming number and organoid size under ENR or WENR conditions are shown. Data are representative of 2 independent experiments. Data are represented as mean ± SD. Statistical analyses were conducted using the Student *t* test and 1-way analysis of variance with the Tukey post-hoc test. ∗*P* < .05; ∗∗*P* < .01. H&E, Hematoxylin and eosin; LI, large intestine; PAS, Periodic acid–Schiff.

We generated IEC-specific LKB1-deficient mice by crossing LKB1^f/f^ with Villin^Cre^ mice (LKB1^ΔIEC^). Despite no difference in body weight between LKB1^ΔIEC^ mice and their littermate controls LKB1^f/f^, LKB1^ΔIEC^ mice exhibited significant increases in the weights of the SI, cecum, and colon, and increased colon lengths compared with LKB1^f/f^ mice (Figure 1*A* and 1*B*). As previously reported by others,^11^ we observed that LKB1^ΔIEC^ caused abnormal development of Paneth and goblet cells. Paneth cells from the SI of LKB1^ΔIEC^ mice exhibited aberrant lysozyme distribution, indicated by diminished and diffuse lysozyme staining (Figure 1*C*). In addition, the quantity of Periodic acid–Schiff-stained goblet cells increased in the SI ileum of LKB1^ΔIEC^ mice compared with controls (Figure 1*D*). Additionally, the mRNA levels of mucin 2 and chromogranin A increased significantly. In contrast, the expression of Paneth cell secretomes such as lysozyme, defensin β1, and Wnt3 decreased in LKB1^ΔIEC^ compared with LKB1^f/f^ mice (Figure 1*E*). Gene Set Enrichment Analysis (GSEA) of the SI revealed a loss of Paneth cell gene signature and a stronger goblet cell gene signature in LKB1^ΔIEC^ mice (Figure 1*F*). Among the transcription factors involved in the differentiation of the secretory cell lineages,^29^ Spdef and Gfi1 were highly expressed in LKB1^ΔIEC^ mice (Figure 1*G*). Furthermore, intestinal stem cell (ISC) marker expression (ie, Lgr5) significantly decreased, whereas major Wnt target gene expression (ie, CD44) was markedly elevated in LKB1^ΔIEC^ mice (Figure 1*H*). Identical phenotypes were observed in the colon (Figure 1*I*). To assess the organoid forming capacity of ISCs, we cultured SI crypts in both ENR and WENR medium. In the ENR medium, SI crypts from LKB1^ΔIEC^ mice showed reduced organoid-forming efficiency and size compared with LKB1^f/f^ mice (Figure 1*J*). However, the quantity of LKB1^ΔIEC^ organoids that formed in the WENR medium was similar to that of LKB1^f/f^ organoids (Figure 1*J*), indicating that the lower organoid formation efficiency of LKB1^ΔIEC^ was caused by the reduction of Wnt3 produced by Paneth cells. Overall, intestinal epithelial LKB1 is involved in ISC proliferation and regulates secretory cell differentiation.

### LKB1^ΔIEC^ Mice Have an Increased Abundance of Akkermansia

**Figure 2:**
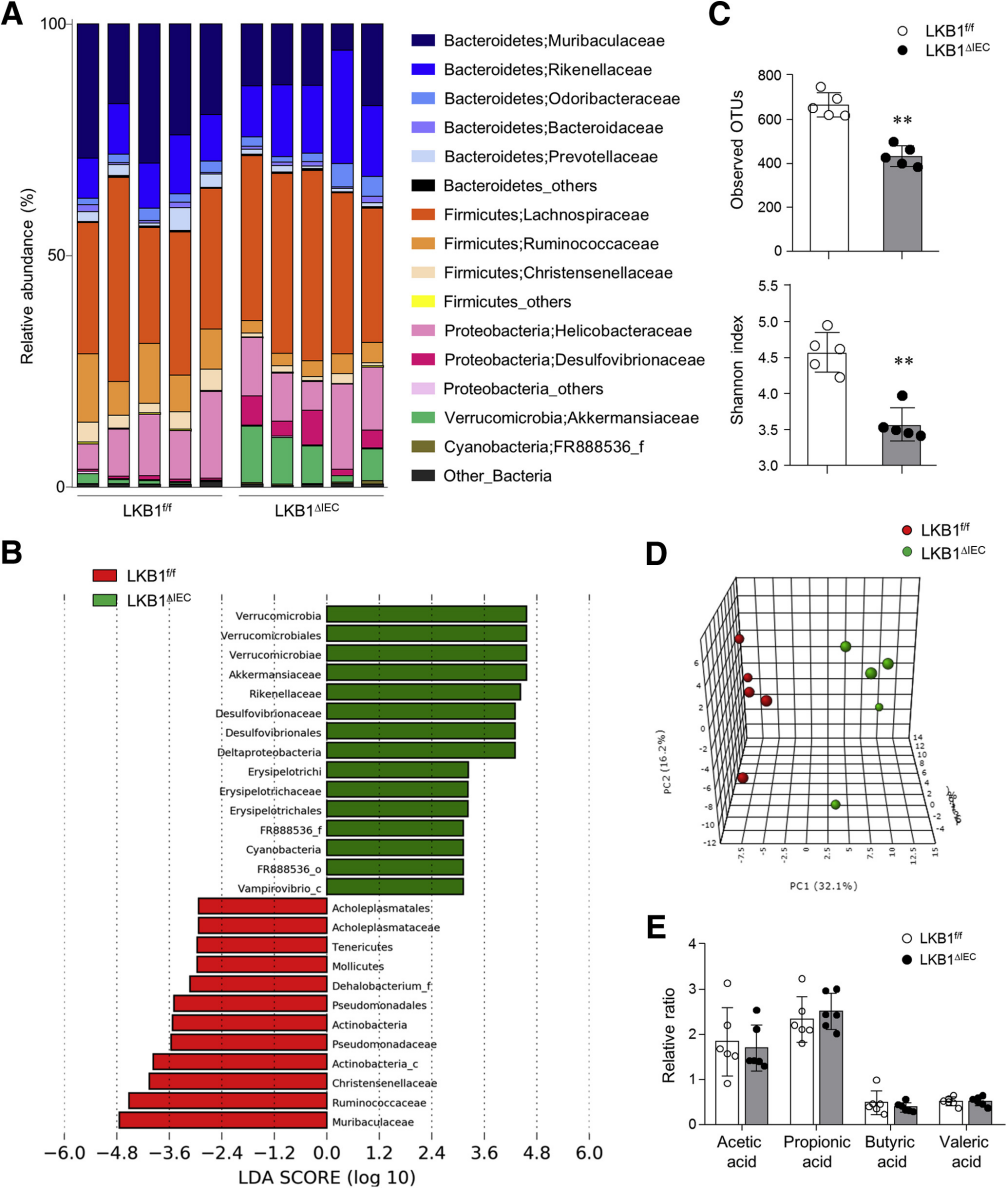
**LKB1**^**ΔIEC**^**mice have an increased abundance of *Akkermansia*.** (*A–D*) 16S rDNA sequencing was performed in the cecum of LKB1^f/f^ and LKB1^ΔIEC^ mice that were housed separately after genotyping (n = 5/group). (*A*) Relative abundance of OTUs at the family level. (*B*) The histogram was generated by the LEfSe analysis. Differentially abundant taxa of microbiota that were enriched in LKB1^f/f^ (*red*) or LKB1^ΔIEC^ (*green*) mice. (*C*) Observed OTUs and Shannon diversity index were determined. (*D*) Principal Coordinates Analysis of weighted UniFrac distances plot showing clustering between the 2 groups. (*E*) Levels of short-chain fatty acids in cecal content were analyzed using LC-MS/MS (n = 6/group). Data are represented as mean ± SD. Statistical analyses were conducted using the Student *t* test. ∗∗*P* < .01. OTUs, Operational taxonomic units.

To address the alteration of gut microbiota in the absence of IEC LKB1, we conducted metagenomic analysis of cecum content. An elevated abundance of *Akkermansia* was observed in the cecum of LKB1^ΔIEC^ mice compared with that of LKB1^f/f^ mice (Figure 2*A* and 2*B*). We speculate that mucin overproduction in LKB1^ΔIEC^ mice contributes to an increase of *Akkermansia*, a mucin-degrading bacterium.^30^ LKB1^ΔIEC^ mice exhibit reduced gut microbial diversity and richness compared with LKB1^f/f^ mice (Figure 2*C*). Furthermore, we observed clear separation of the bacterial communities between LKB1^f/f^ and LKB1^ΔIEC^ mice (Figure 2*D*). Despite a significant change in microbiota composition, levels of short-chain fatty acids in the cecum content of LKB1^ΔIEC^ mice were comparable to LKB1^f/f^ mice (Figure 2*E*). We used co-housing LKB1^f/f^ and LKB1^ΔIEC^ mice to minimize the influence of gut microbiota in further studies.

### Intestinal LKB1 Loss Accelerated BA Synthesis and Secretion

**Figure 3:**
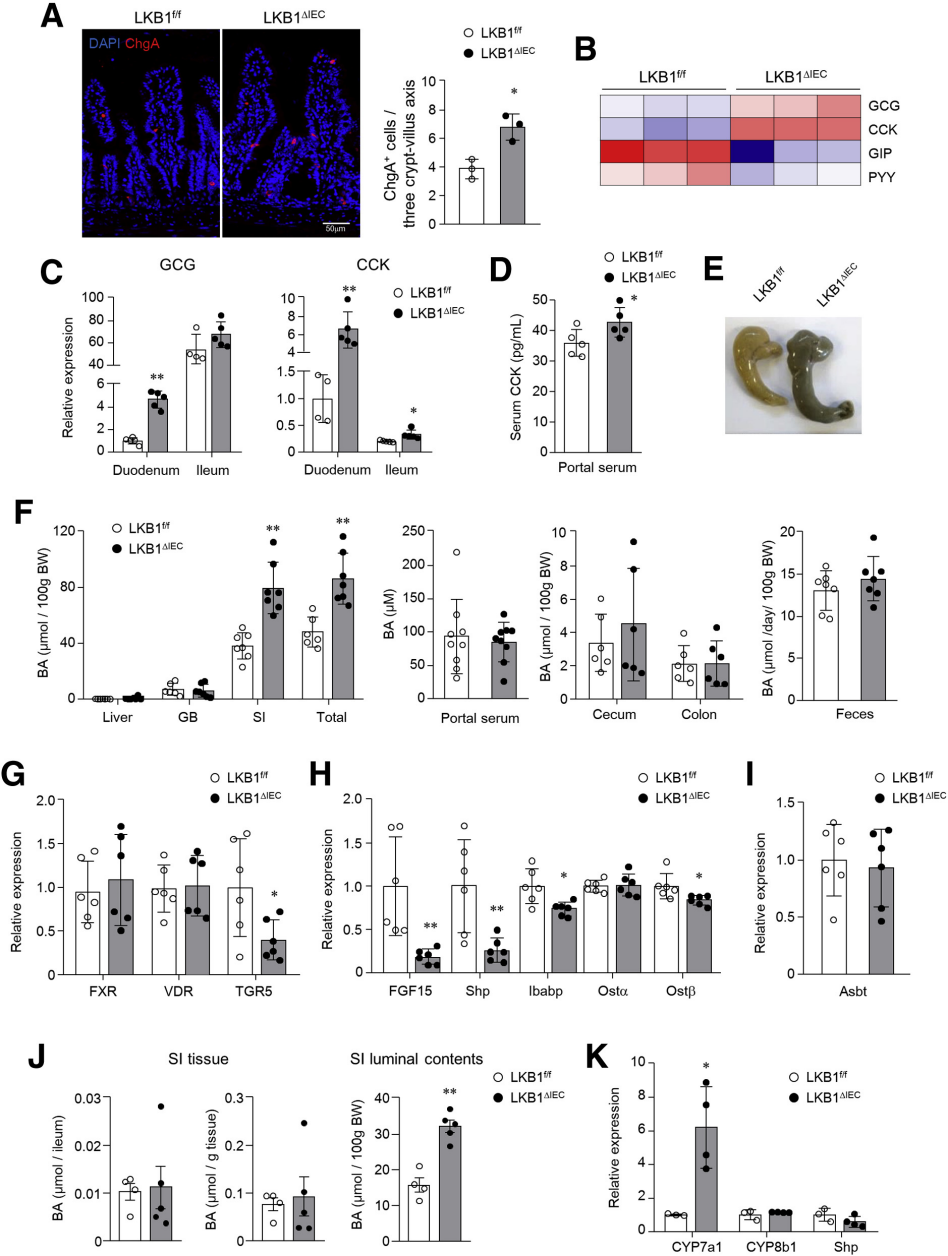
**Bile acid pool size increased in LKB1**^**ΔIEC**^**mice.** (*A*) Representative images of immunofluorescence staining for chromogranin A (*red*) and quantification of enteroendocrine cells per 3 crypt-villus axis in the SI (n = 3/group). Bar = 50 μm. (*B*) The heat map of differentially expressed genes (fold change ≥1.5; *P* < .05) of the gut hormones based on RNA-seq data of the SI ileum from LKB1^f/f^ and LKB1^ΔIEC^ mice. (*C*) mRNA levels of GCG and CCK in the duodenum and ileum (n = 4–5/group). (*D*) CCK level was measured in serum from portal venous blood (n = 5/group). (*E*) The representative image of cecum. (*F*) Total BA concentration in the liver, GB, SI, and total pool (liver + GB + SI) from mice fasted for 4 hours (n = 6–7/group). BA levels in the portal serum, cecum and colon, and feces (n = 6–9/group). mRNA expression levels of BA receptors (*G*), FXR target genes (*H*), and BA transporter (Asbt; *I*) in the SI ileum were measured by qPCR (n = 6/group). (*J*) Total BA concentration in the SI ileum tissue and SI luminal contents (n=4–5/group). (*K*) Gene expression of enzymes involved in BA synthesis in the liver was assessed (n = 3–4/group). Data are represented as mean ± SD. Statistical analyses were conducted using the Student *t* test. ∗*P* < .05; ∗∗*P* < .01. GB, Gallbladder; GCG, glucagon.

In addition to the hyper goblet cell phenotype, we observed increased expression of the chromogranin A enteroendocrine cell marker in the SI of LKB1^ΔIEC^ mice (Figures 1*E* and 3*A*). Furthermore, among various peptide hormones in the RNA-seq data, higher expression of glucagon and cholecystokinin (CCK) but lower expression of glucose-dependent insulinotropic peptide and peptide YY were observed in the SI of LKB1^ΔIEC^ mice compared with LKB1^f/f^ mice (Figure 3*B*). Predominant mRNA levels of glucagon and CCK were determined in the duodenum and ileum of LKB1^ΔIEC^ mice by reverse transcription- polymerase chain reaction (PCR) (Figure 3*C*). Elevated CCK levels in the portal serum (Figure 3*D*) and enlarged dark green-brown cecum were also observed in LKB1^ΔIEC^ mice (Figures 3*E*). Previous studies reported that CCK promotes gallbladder contraction,^31^ and bile pigment renders the color of the stool dark.^32^ Therefore, we further analyzed total BA levels in the liver, gallbladder, SI, portal serum, cecum, and colon from mice fasted for 4 hours. We unexpectedly found that they were significantly elevated in the SI of LKB1^ΔIEC^ mice, along with an increase in total BA pool size, defined as the total BA within enterohepatic circulation (the sum of BA in the liver, gallbladder, and SI) (Figure 3*F*). In contrast, no significant changes in BA was noted in the hepatic, gallbladder, portal serum, cecum, colon, and fecal extracts (Figure 3*F*), indicating that deletion of LKB1 increases BA accumulation in the SI without affecting excretion. BA level is controlled by a negative feedback loop within the liver and intestine in response to changes in BA levels.^33^ Hence, we evaluated gene expression involved in the intestinal BA feedback pathway to identify the factors that increase BA pool size in LKB1^ΔIEC^ mice. Among the BA receptors, the expression level of TGR5, but not FXR and vitamin D receptor, decreased significantly in the SI ileum of LKB1^ΔIEC^ mice (Figure 3*G*). Despite no changes in FXR expression, mRNA levels of its target genes (ie, FGF15, Shp, Ibabp, and Ostβ) involved in BA synthesis and transport were lower in LKB1^ΔIEC^ mice than in LKB1^f/f^ mice (Figure 3*H*). On the other hand, there was no change in Asbt expression, which is responsible for ileal BA absorption (Figure 3*I*). A previous study reported that reduced expression of Ostα/β, a basolateral transporter mediating the transport of BA from IECs to the lamina propria, was involved in the accumulation of BA in IECs and altered intestinal morphology.^34^ However, we observed that increased BA of the SI in LKB1^ΔIEC^ mice primarily accumulated in the SI lumen but not in the SI tissue, including the IECs (Figure 3*J*). Consistent with reduced FGF15 expression, we found a significant increase in hepatic CYP7a1 in LKB1^ΔIEC^ mice (Figure 3*K*). We can therefore infer that intestinal deletion of LKB1 causes interrupted FGF15-mediated feedback control of CYP7a1, resulting in enhanced BA synthesis.

### LKB1 Deletion Reduced FGF15/19 Secretion in an FXR-independent Manner

**Figure 4:**
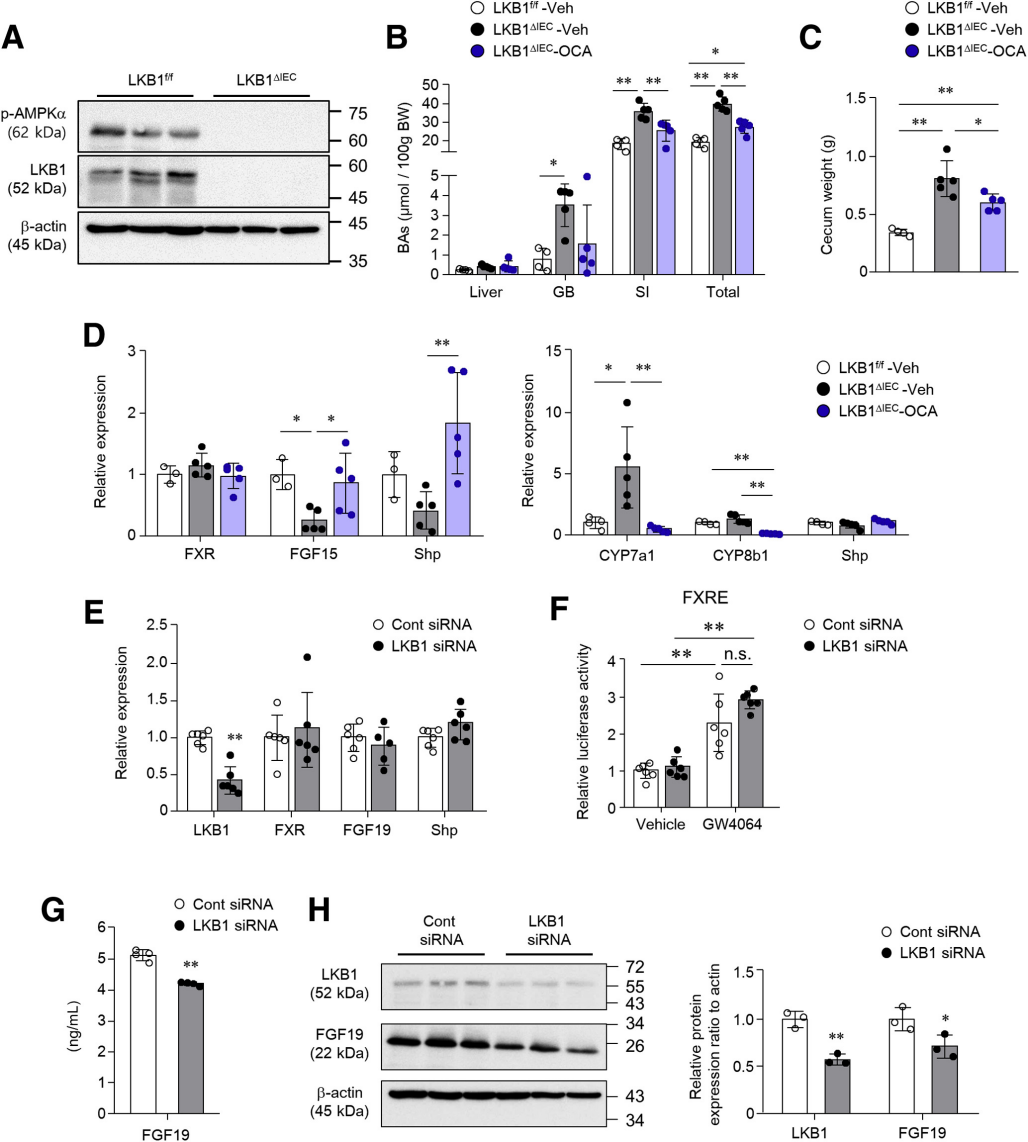
**LKB1 depletion in human IECs decreased FGF19 protein level in an FXR-independent manner.** (*A*) Western blot analysis of the SI tissues in LKB1^f/f^ and LKB1^ΔIEC^ mice. (*B–D*) Mice were administered vehicle (10% DMSO and 90% sunflower oil) or OCA (30 mg/kg) by oral gavage (over 4 consecutive days and 4 hours before euthanasia) (n = 4–5/group) and euthanized after fasting for 4 hours. (*B*) BA levels in the liver, GB, SI, and (*C*) cecum weight were measured. (*D*) mRNA expression levels in the SI ileum (*left*) and liver (*right*). (*E*) Caco2 cells were transfected with 10 nM scrambled control or LKB1 siRNA. Expression levels of LKB1, FXR, FGF19, and Shp were measured by qPCR at 48 hours after transfection (n = 6/group). (*F*) Caco2 cells transfected with FXRE luciferase reporter and control or LKB1 siRNA (10 nM) were treated with GW4064 (5 μM) or vehicle at 24 hours post-transfection. After 24 hours of treatment, luciferase activity was measured (n = 6/group). (*G and H*) After siRNA (25 nM) transfection in HT-29 cells for 48 hours, FGF19 protein level was measured with cell culture supernatants using ELISA (*G*) (n = 4/group), and with cell extracts by Western blot analysis (*H*). Data are representative of 2 independent experiments. Data are represented as mean ± standard deviation (SD). Statistical analyses were conducted using the Student *t* test and 1-way analysis of variance with the Tukey post-hoc test. ∗*P* < .05; ∗∗*P* < .01. ELISA, Enzyme-linked immunosorbent assay; FXRE, FXR response element; GB, gallbladder; siRNA, small interfering RNA.

Because activated AMPK prevented FXR transcriptional activity through its phosphorylation, silencing of AMPK enhanced FXR and FGF19 expression.^35^ However, despite decreased AMPKα phosphorylation (Figure 4*A*) and increased circulating total BA levels, the proportion of FXR downstream genes decreased in LKB1^ΔIEC^ mice. We treated LKB1^ΔIEC^ mice with an FXR agonist (ie, obeticholic acid [OCA]) to determine whether FXR-mediated signal transduction is functional. Vehicle (10% DMSO and 90% sunflower oil) or OCA was orally administered for 5 days. OCA treatment significantly reduced BA levels in the SI and BA pool size (Figure 4*B*), and diminished cecum weight (Figure 4*C*) of LKB1^ΔIEC^ mice. Moreover, it led to increased expression of FGF15 and Shp in the SI and decreased expression of CYP7a1 and CYP8b1 in the liver (Figure 4*D*), suggesting that OCA stimulates FGF15/19 production and inhibits abnormal BA production in LKB1^ΔIEC^ mice. Overall, these results demonstrated that the FXR pathway is intact in LKB1^ΔIEC^ mice. To explore whether LKB1 knockdown directly affects the FXR pathway and FGF15/19 expression in IECs, we transfected human IECs (ie, Caco2 and HT-29 cell lines) with LKB1 siRNA. siRNA-mediated LKB1 knockdown (up to 50%–70%) had no effect on the mRNA levels of FXR, FGF19, and Shp (Figure 4*E*). Additionally, the luciferase reporter assay revealed that LKB1 depletion did not interrupt FXR activation in the presence of the FXR agonist (ie, GW4064) (Figure 4*F*), indicating that LKB1 does not directly modulate the FXR signaling pathway. However, we observed significantly lower FGF19 protein levels in the culture medium treated with LKB1 siRNA treatment (Figure 4*G*). Western blot analysis confirmed a silencing of LKB1 concomitant with dampened FGF19 protein level (Figure 4*H*), indicating that FGF19 protein level is regulated by LKB1 depletion in a cell-autonomous and transcription-independent manner. Overall, these results suggest that LKB1 deletion resulted in downregulation of FGF15/19 secretion in an FXR-independent manner.

### LKB1^ΔIEC^ Mice Had an Accumulation of Conjugated BA and Decreased BSH-producing Bacteria

**Figure 5:**
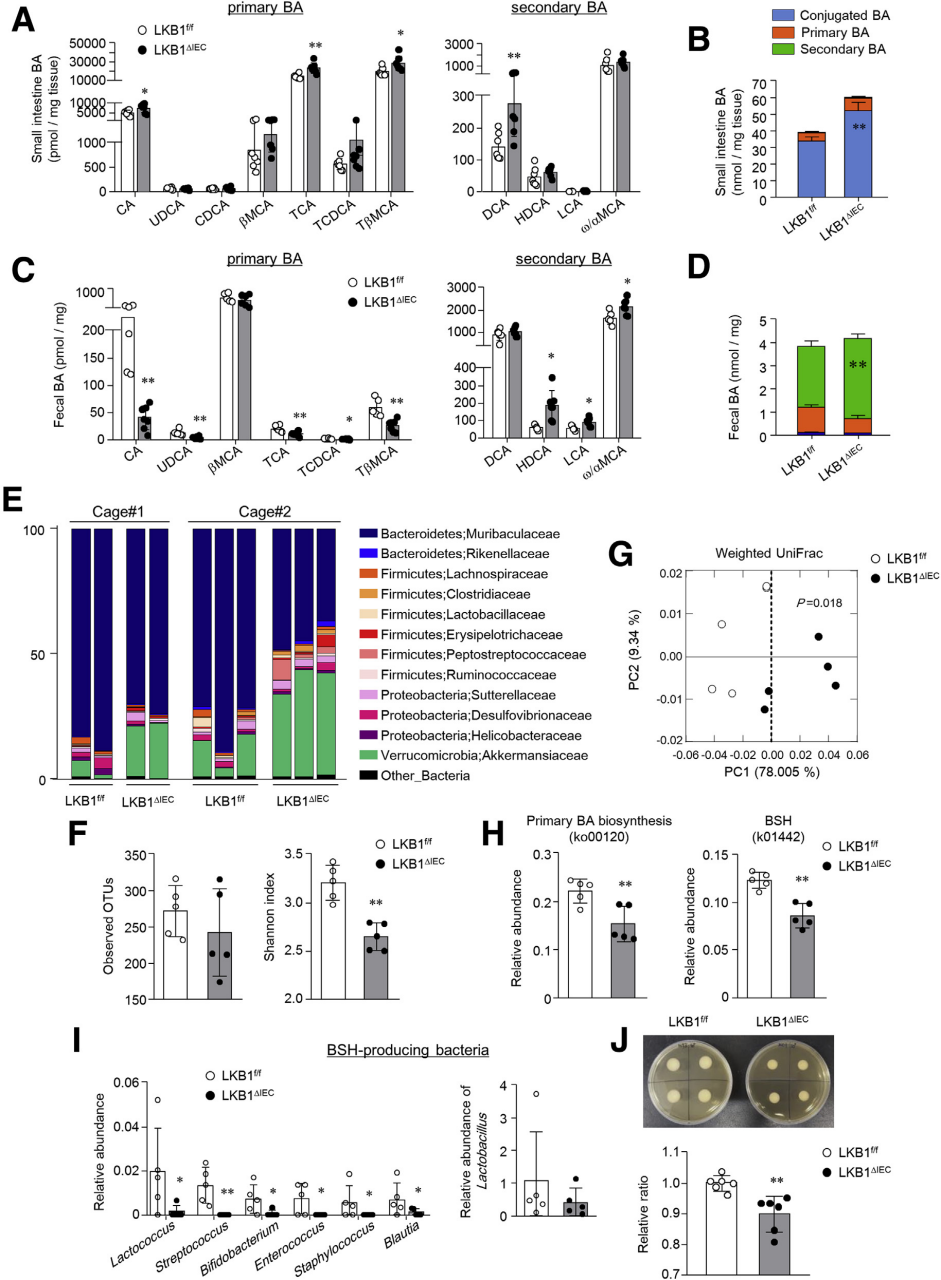
**LKB1**^**ΔIEC**^**mice had increased levels of conjugated BAs and reduced BSH-producing bacteria in the SI.** (*A*) BA profile of the SI and (*B*) the levels of conjugated, primary, and secondary BAs (n = 7/group). (*C and D*) BA profile of the feces (n = 5–7/group). (*E–I*) 16S rDNA sequencing in the SI of LKB1^f/f^ and LKB1^ΔIEC^ mice housed in 2 different cages (n = 5/group) was performed. (*E*) Relative abundance of OTUs at family levels. (*F*) Observed OTUs and the Shannon diversity index were determined. (*G*) Principal coordinate analysis of weighted UniFrac distances was conducted. (*H*) Relative abundance of genes involved in primary BA biosynthesis (ko00120) and BSH (k01442) were estimated by PICRUSt prediction analysis. (*I*) Relative abundance of BSH-producing genera was calculated. (*J*) The BSH activity of SI content was examined using an agar plate assay. The size of white precipitate halos was measured (n = 6/group). Data are representative of 2 independent experiments and represented as mean ± standard deviation (SD). Statistical analyses were conducted using the Student *t* test. ∗*P* < .05; ∗∗*P* < .01. OTUs, Operational taxonomic units.

In vitro assay revealed that LKB1 loss in IECs resulted in no apparent effects on FXR signaling; however, LKB1^ΔIEC^ mice exhibited a marked reduction in FXR target genes. Hence, we explored other factors that can inhibit FXR signaling in LKB1^ΔIEC^ mice. Given the regulatory effects of BA composition on FGF15/19 production as an agonist or antagonist of FXR, we proceeded with BA composition analysis. LKB1^ΔIEC^ mice had higher levels of cholic acid (CA), taurocholic acid (TCA), T-βMCA, and deoxycholic acid (DCA) in the SI than LKB1^f/f^ mice (Figure 5*A*). The increase of BA in the SI of LKB1^ΔIEC^ mice was primarily due to elevated levels of conjugated BA (Figure 5*B*). Furthermore, a lower and higher proportion of primary and secondary BA, respectively, were detected in the feces of LKB1^ΔIEC^ mice compared with LKB1^f/f^ mice (Figure 5*C* and 5*D*). These results demonstrate that LKB1 depletion in IECs alters BA profiles, coinciding with an increase in BA pool size. Next, given that microbiota affect BA profiles, we analyzed their composition in the SI. Despite co-housing LKB1^ΔIEC^ mice with their littermate controls, *Akkermansia* were significantly enriched in the SI of LKB1^ΔIEC^ mice (Figure 5*E*). Although there was no difference in the observed number of species, Shannon diversity was considerably lower in these mice compared with LKB1^f/f^ mice (Figure 5*F*). Beta diversity analysis further revealed significant differences in bacterial composition between LKB1^f/f^ and LKB1^ΔIEC^ mice (Figure 5*G*). The functional capacity of the gut microbiome, estimated with PICRUSt prediction analysis based on 16S rRNA profiles, demonstrated a significantly lower abundance of genes associated with primary BA biosynthesis and BSH in LKB1^ΔIEC^ mice (Figure 5*H*). In this regard, LKB1^ΔIEC^ mice harbored a robustly lower abundance of several microbial genera containing BSH,^36^^,^^37^ including *Lactococcus*, *Streptococcus*, *Bifidobacterium*, *Enterococcus*, *Staphylococcus*, *Blautia*, and a slightly reduced level of *Lactobacillus* (Figure 5*I*). Additionally, BSH activity had reduced substantially in the content of these mice (Figure 5*J*). These results suggest that reduction of BSH-producing bacteria in LKB1^ΔIEC^ mice leads to altered BA metabolism, particularly the accumulation of T- βMCA, which acts as an FXR antagonist.

### LKB1-depleted IECs Exhibit Impaired Retinol Metabolism, Affecting FGF15/19 Production

**Figure 6:**
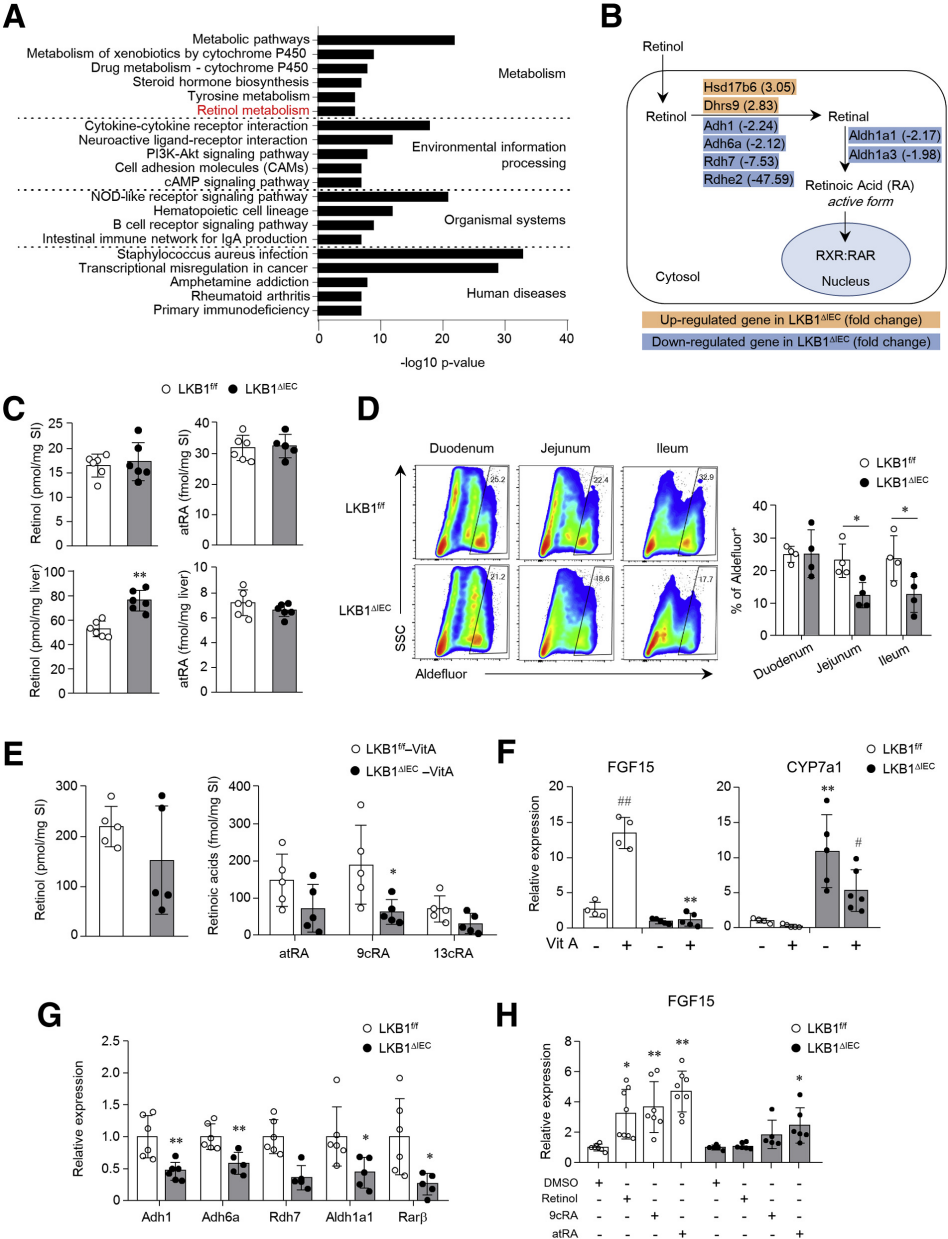
**LKB1**^**ΔIEC**^**mice showed impaired retinol conversion to retinoic acids in the SI.** (*A*) The top 20 most enriched KEGG pathways of differentially expressed genes based on RNA-seq data of the SI ileum from LKB1^f/f^ and LKB1^ΔIEC^ mice (n = 3 / group). (*B*) Partial scheme of retinol metabolism focuses on RA synthesis from retinol. Differentially expressed genes along the pathway are shown in the boxes. *Blue* and *red* colors indicate downregulation and upregulation in the SI ileum from LKB1^ΔIEC^ mice compared with LKB1^f/f^ mice, respectively. The numbers in brackets indicate fold change in RNA-seq (n = 3/group). (*C*) Quantification of retinol and atRA in the whole SI and liver of LKB1^f/f^ and LKB1^ΔIEC^ mice was conducted using LC-MS/MS (n = 6/group). (*D*) Aldehyde dehydrogenase activity in IECs was detected using the Aldefluor assay and measured by flow cytometry (n = 4/group). (*E and F*) LKB1^f/f^ and LKB1^ΔIEC^ mice were orally treated with vehicle or 100 mg/kg retinyl palmitate (Vit A) for 16 hours. (*E*) Quantities of retinol and RAs, including atRA, 9cRA, and 13-cis RA (13cRA), were measured in ileum tissues using LC-MS/MS (n = 5/group). (*F*) Gene expression of FGF15 in the ileum and CYP7a1 in the liver was measured (n = 5–6/group). (*G*) mRNA expression levels of enzymes associated with retinol metabolism and Rarβ, a gene positively regulated by RA, in the SI organoids were measured by qPCR (n = 5–6/group). (*H*) SI organoids from LKB1^f/f^ and LKB1^ΔIEC^ mice were treated with DMSO, retinol, 9cRA, or atRA for 3 days. Fgf15 expression was measured by qPCR (n = 7/group). Data are representative of 2 independent experiments. Data are represented as mean ± standard deviation (SD). Statistical analyses were conducted using the Student *t* test and 1-way analysis of variance with the Tukey post-hoc test. ∗*P* < .05; ∗∗*P* < .01 compared with the control mice of the same treatment group. ^#^*P* < .05; ^##^*P* < .01 compared with the vehicle of the same genotype. DEGs, Differentially expressed genes.

Retinoic acid (RA) regulates FGF15/19 expression through binding to the retinoid X receptor (RXR),^38^ which forms a heterodimer with FXR.^39^ The KEGG pathway enrichment analysis revealed that differentially expressed genes between the SI of LKB1^f/f^ and LKB1^ΔIEC^ mice were significantly enriched in retinol metabolism (Figure 6*A*). Moreover, genes involved in the biosynthesis of RA from retinol, such as Adh1, Adh6a, Rdh7, Aldh1a1, and Aldh1a3, were downregulated in the SI of LKB1^ΔIEC^ mice compared with LKB1^f/f^ mice (Figure 6*B*). We then quantified the amount of retinol and RA in the SI and liver using liquid chromatography (LC)-mass spectrometry (MS/MS). Although retinol and all-trans retinoic acid (atRA) levels were similar in the SI of LKB1^f/f^ and LKB1^ΔIEC^ mice, markedly higher quantities of retinol, but not atRA, were detected in the liver of LKB1^ΔIEC^ mice (Figure 6*C*). This result provides evidence to support the alterations in retinol metabolism in LKB1^ΔIEC^ mice. Aldehyde dehydrogenases, crucial enzymes that convert retinal to RA,^40^ were observed in lower levels in the jejunum and ileum of LKB1^ΔIEC^ mice than in LKB1^f/f^ mice (Figure 6*D*). Administration of vitamin A resulted in a substantial reduction in 9-cis retinoic acid (9cRA) in the ileum tissue of LKB1^ΔIEC^ mice compared with LKB1^f/f^ mice (Figure 6*E*), indicating reduced RA biosynthesis in the ileum of LKB1^ΔIEC^ mice. Furthermore, administration of vitamin A led to increased FGF15 expression in LKB1^f/f^ mice but not in LKB1^ΔIEC^ mice (Figure 6*F*). In contrast, high expression of hepatic CYP7a1 was dampened by vitamin A treatment in LKB1^ΔIEC^ mice (Figure 6*F*). As previously reported,^38^ this may extend from additional vitamin A mechanisms in CYP7a1 suppression, not through activation of the intestinal FXR-FGF15/19 signaling pathway. We also investigated the potential for retinol and RA to stimulate FGF15 expression in LKB1-depleted IECs using intestinal organoids. Intestinal organoids from LKB1^ΔIEC^ mice expressed lower level of genes related to RA biosynthesis than those from LKB1^f/f^ mice (Figure 6*G*). Whereas retinol, 9cRA, or atRA activated FGF15 expression in SI organoids from LKB1^f/f^ mice, only RAs elevated FGF15 expression in LKB1^ΔIEC^ mice (Figure 6*H*). These results indicate that impaired retinol conversion to RA may be an underlying mechanism for disrupting FGF15/19 signaling in the absence of LKB1.

### HFD-fed LKB1^ΔIEC^ Mice Revealed Improved Glucose Tolerance and Elevated Energy Expenditure

**Figure 7:**
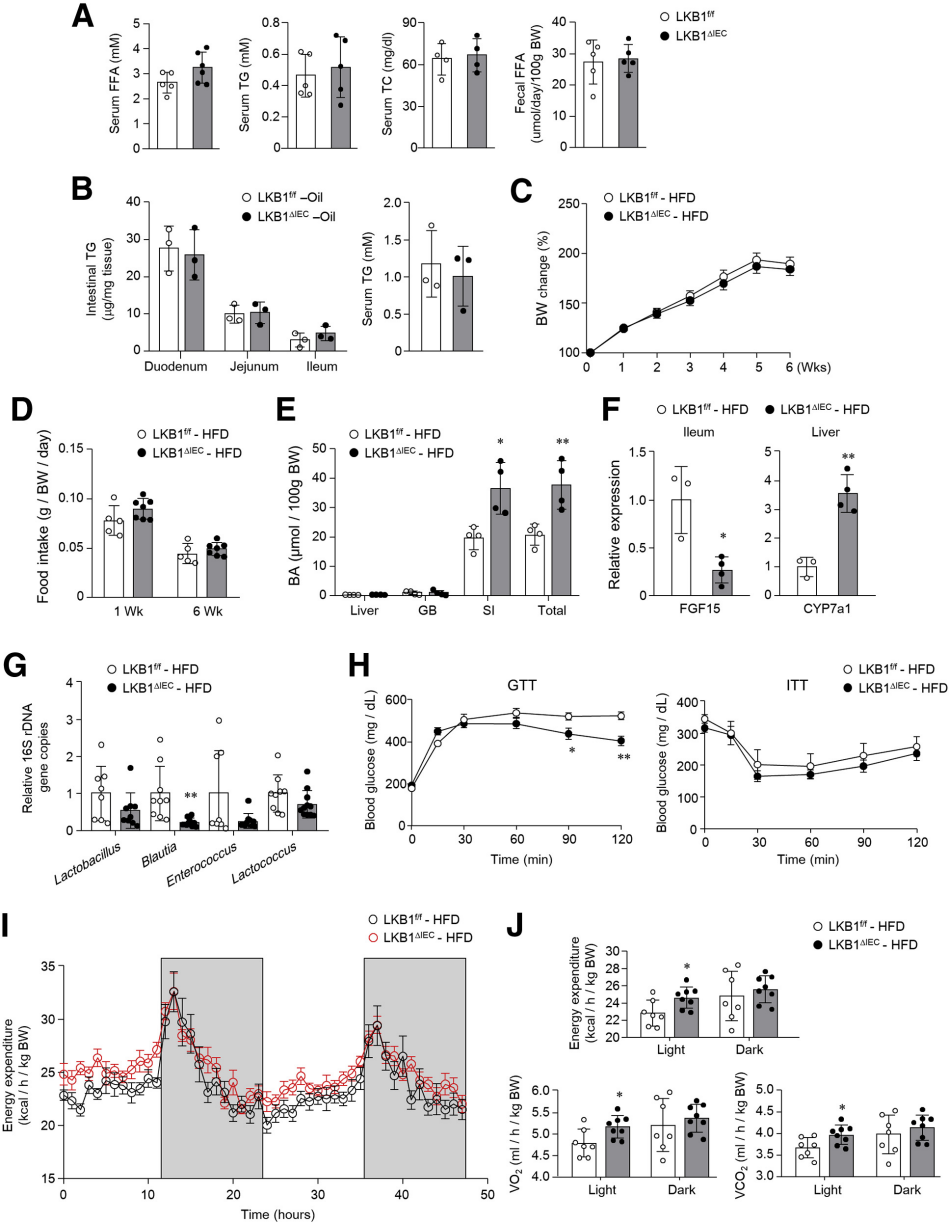
**LKB1**^**ΔIEC**^**mice on a high-fat diet exhibit improved glucose intolerance and increased energy expenditure.** (*A*) Serum levels of FFA, TG, TC, and fecal FFA level were assessed in 8-week-old mice using colorimetric assay kits (n = 4–6/group). (*B*) TG concentrations in the SI and serum were measured at 4 hours after oral gavage of olive oil (10 μl/g of body weight) (n = 3/group). (*C*) Body weights of LKB1^f/f^ (n = 9) and LKB1^ΔIEC^ (n = 11) mice fed an HFD for 6 weeks. (*D*) Daily food intake for HFD-fed LKB1^f/f^ (n = 5) and LKB1^ΔIEC^ (n = 7) mice at 1 and 6 weeks of HFD. (*E*) BA concentration in the liver, GB, SI, and total pool (liver + GB + SI) (n = 4/group). (*F*) mRNA expression levels in the SI ileum (*left*) and liver (*right*) (n = 3–4/group). (*G*) Quantification of BSH-producing bacteria 16s rDNA gene copies in the cecal contents by qPCR (n = 8–11/group). (*H*) GTT and ITT were performed on mice fed an HFD for 6 and 8 weeks, respectively (n = 8/group). (*I and J*) Energy expenditure, O_2_ consumption, and CO_2_ production rate were measured by the Comprehensive Laboratory Animal Monitoring System after 7 weeks of HFD feeding. Bar graphs indicate the average value during the light and dark cycle (n = 7–8/group). Data are representative of two independent experiments and represented as mean ± SD. Statistical analyses were conducted using the Student *t* test and 2-way analysis of variance with the Bonferroni post-hoc test. ∗*P* < .05; ∗∗*P* < .01. FFA, Free fatty acids; GB, gallbladder; GTT, glucose tolerance test; ITT, insulin tolerance test; TC, total cholesterol; TG, triglyceride.

Because BA is synthesized from cholesterol and promotes lipid absorption,^17^ mutation of FXR, FGF15/19, and CYP7a1 led to altered lipid profiles.^41^, ^42^, ^43^ Despite elevated levels of circulating BA (Figure 3*F*), similar levels of free fatty acids, triglyceride, and total cholesterol in the serum, and of free fatty acids in the feces were found in LKB1^f/f^ and LKB1^ΔIEC^ mice (Figure 7*A*). Moreover, no difference in triglyceride levels in the SI and serum from both mice after olive oil feeding (Figure 7*B*) indicates that postprandial fat absorption may not be altered by LKB1 deletion. It has been suggested that intestinal FXR signaling may be involved in whole-body metabolism^24^^,^^44^; we therefore investigated the effects of intestinal LKB1 depletion on host metabolism. Bodyweight gain and food intake were similar in HFD-fed LKB1^f/f^ and LKB1^ΔIEC^ mice (Figure 7*C* and 7*D*). Under HFD conditions, LKB1^ΔIEC^ mice retained elevated BA levels in the SI and total pool (Figure 7*E*). In addition, HFD-fed LKB1^ΔIEC^ mice showed decreased expression of ileal FGF15 but upregulated expression of hepatic CYP7a1 compared with HFD-fed LKB1^f/f^ mice (Figure 7*F*). BSH-producing bacteria show a tendency to decrease in HFD-fed LKB1^ΔIEC^ mice (Figure 7*G*). Of note, HFD-fed LKB1^ΔIEC^ mice exhibited faster glucose clearance than LKB1^f/f^ mice in the glucose tolerance test, but similar insulin sensitivity was observed in both species (Figure 7*H*). Metabolic analysis revealed higher energy expenditure, O_2_ consumption, and CO_2_ production during the light cycle in HFD-fed LKB1^ΔIEC^ mice compared with HFD-fed LKB1^f/f^ mice, suggesting that resting metabolic rate increased in LKB1^ΔIEC^ mice, but did not affect the dark cycle (Figure 7*I* and 7*J*). Overall, the ablation of intestinal LKB1 leads to the improvement in HFD-induced glucose tolerance and increased energy expenditure.

## Discussion

In this study, we found that LKB1 loss in IECs caused a reduction of FGF15/19 production, resulting in an elevation of hepatic CYP7a1 and BA pool size. LKB1 depletion directly reduced FGF15/19 secretion in an FXR-independent manner. Additionally, FGF15/19 production was inhibited through increased T-βMCA, decreased BSH-producing bacteria, and impaired retinol metabolism in LKB1^ΔIEC^ mice. These results indicate that LKB1 signaling in IECs is indispensable in maintaining host homeostasis through BA metabolism.

LKB1 deficiency in IECs caused altered maturation and differentiation of secretory lineage cells. A recent study noted that LKB1 depletion increased the expression of Atoh1, essential for secretory cell formation, via pyruvate dehydrogenase kinase 4.^45^ We observed elevated expression of Atoh1 downstream genes Gfi1 and Spdef, which direct secretory cell fates to goblet and Paneth cells.^46^^,^^47^ Although goblet cell hyperplasia was observed throughout the whole intestinal tract, morphological abnormalities were observed in Paneth cells in the crypt, including increased size and containing mucins. Previous reports have noted that these cells are ‘intermediate’ goblet and Paneth cells.^11^^,^^48^ The overexpression of Spdef-directed goblet and Paneth progenitors into terminal differentiation of goblet cells and caused a decrease in Paneth cells.^49^ This may therefore explain the abnormal regulation of secretory lineage cells in LKB1^ΔIEC^ mice.

We primarily focused on dysregulated BA homeostasis in LKB1^ΔIEC^ mice. The regulatory roles of intestinal SIRT1 and AMPK, up and downstream to LKB1, respectively, in BA homeostasis have been previously described.^35^^,^^50^ Intestinal SIRT1-deleted mice had elevated BA synthesis and CYP7a1 expression, but exhibited normal total BA pool size and increased fecal BA excretion.^50^ They demonstrated that SIRT1 loss in the intestine impaired the hepatocyte nuclear factor-1α signaling pathway and reduced expression of downstream genes such as FXR and Asbt.^50^ We observed that LKB1 depletion did not affect the expression levels of these genes, indicating that LKB1 does not influence the hepatocyte nuclear factor-1α signaling pathway. Furthermore, LKB1^ΔIEC^ mice showed increased BA pool size, but normal fecal BA levels. These findings were in line with a previous study that noted that germ-free mice also exhibited an elevated BA pool size and reduced excretion.^25^ Moreover, treatment of probiotic strains with high BSH activity enhanced BA deconjugation and fecal excretion.^51^ We therefore speculated that increased BA pool without changes to BA excretion results from predominantly conjugated BAs in the SI of LKB1^ΔIEC^ mice, which are primarily reabsorbed in the terminal ileum by Asbt.^52^

In vitro study revealed that acute depletion of LKB1 caused a significant reduction of FGF15/19 protein levels in an FXR-independent manner. Diet1 has been shown to control FGF15/19 levels via an FXR-independent pathway.^53^ The authors demonstrated co-localization and co-immunoprecipitation of Diet1 and FGF15/19 proteins, allowing for posttranslational regulation.^53^ Therefore, we speculate that LKB1 could regulate FGF15/19 levels, in part, through a posttranslational mechanism.

FXR target genes were largely reduced in vivo, which suggests that other factors must indirectly inhibit FGF15/19. An increase of T-βMCA in the SI of LKB1^ΔIEC^ mice contributes to the inhibition of FXR activation. We observed a lower abundance of BSH-producing bacteria, involved in the deconjugation of conjugated BAs, in LKB1^ΔIEC^ mice. LKB1 ablation caused a reduction in microbial diversity and community changes compared with control mice, despite co-housing. The luminal environment of LKB1^ΔIEC^ mice, such as altered defensin, mucin, or BA production, which can regulate bacterial overgrowth and composition, may be a contributor.^54^, ^55^, ^56^ In addition, a high concentration of CA and DCA inhibits the growth of *Bifidobacterium* and *Lactobacillus* by disturbance of membrane integrity.^57^ Thus, we assumed that a reduced abundance of these bacteria might be partially affected by the higher CA and DCA levels in the SI of LKB1^ΔIEC^ mice.

Disturbed retinol metabolism in LKB1-deleted IECs also can affect FXR downstream gene expression through RXR. Decreased 9cRA and FGF15 expression in vitamin A-treated LKB1^ΔIEC^ mice suggest that the synthesis of biologically active metabolites (ie, RAs) from vitamin A was inhibited by LKB1 loss. We believe that this study is the first to examine the regulatory role of LKB1 in retinol metabolism. Previous research has shown through transcriptome analysis that differentially expressed genes between cells with and without LKB1 deletion were significantly enriched in RXR-mediated signaling pathways.^58^ Overall, our results indicate the potential function of LKB1 on RXR-dependent pathways activated by RA.

A recent study revealed that HFD-fed AMPK^ΔIEC^ mice had no differences in glucose tolerance and energy expenditure compared with control mice.^59^ We therefore speculate that metabolic improvements of LKB1^ΔIEC^ mice were in an AMPK-independent manner. Previous studies demonstrated that FXR signaling and BA pool size contributes to metabolic changes. Mice treated with an FXR agonist exhibited a decreased BA pool size and energy expenditure, causing susceptibility to HFD-induced obesity.^60^ However, mice treated with an intestinal FXR inhibitor and transgenic mice overexpressing CYP7a1 had increased BA pool size and energy expenditure, and were thus were highly resistant to HFD-induced obesity.^61^^,^^62^ Hence, metabolic changes in HFD-fed LKB1^ΔIEC^ mice resulted from an increased BA pool size. However, despite the increase in BA pool size, LKB1^ΔIEC^ mice were not resistant to weight gain within 8 weeks of HFD feeding. Because LKB1 regulates energy metabolism, other factors besides BA levels might have affected the development of obesity in LKB1^ΔIEC^ mice.

This study demonstrates a regulatory role for intestinal LKB1 on BA synthesis through control of FGF15/19 signaling. Additionally, LKBI may have a preventive effect on RA metabolism. Our findings revealed a new function of intestinal LKB1 on systemic BA homeostasis. Because LKB1, AMPK, and SIRT1 are attractive targets for metabolic disorders, their reciprocal interactions to maintain BA homeostasis should be further elucidated.

## Methods

### Animal Experiments

All animal experiments were approved by the Institutional Animal Care and Use Committee of Asan Institute for Life Sciences (Approval No. 2020-12-131), and studies were conducted in accordance with the approved guidelines and regulations. LKB1^ΔIEC^ mice were generated by crossing LKB1^f/f^ and Villin^Cre^ mice (Jackson Laboratory, Bar Harbor, ME). LKB1^ΔIEC^ mice were co-housed with their control littermates (LKB1^f/f^) after genotyping. All mice were maintained in groups of 2 to 5 in cages under specific pathogen-free conditions and received sterilized food and water ad libitum. All experiments were performed with sex- and age-matched 6- to 12-week-old mice. Mice were fasted 4 hours before euthanasia, and blood and tissues were collected under anesthesia with a mixture of ketamine (100 mg/kg) and xylazine (20 mg/kg). For HFD experiments, LKB1^f/f^ and LKB1^ΔIEC^ mice were fed a HFD (60% of total calories from fat) for 8 weeks. OCA (AdipoGen, San Diego, CA), dissolved in 10% DMSO (BioLife Solution, Bothell, WA) and 90% sunflower oil (Sigma Aldrich, St. Louis, MO), was administered by oral gavage at a dose of 30 mg/kg for 5 days. Vitamin A (retinyl palmitate, Sigma Aldrich, 100 mg/kg) in sunflower oil was administered by oral gavage at the beginning and end of the dark cycle (16 and 4 hours before death), described previously.^38^

### Histological and Immunofluorescence Staining

The SI and colon sections were fixed in 4% paraformaldehyde and embedded in paraffin. For histological analysis, tissues were stained with hematoxylin and eosin or Periodic acid–Schiff. For immunofluorescence staining, sections were deparaffinized with xylene and rehydrated in a series of alcohol baths. Heat-induced antigen retrieval was performed in sodium citrate buffer (10 mM, pH 6.0) at 97°C for 10 minutes. After cooling, tissue sections were permeabilized with 0.5% Triton X-100 for 10 minutes, and blocked with 5% bovine serum albumin in phosphate-buffered saline (PBS) for 1 hour, followed by staining with primary antibodies overnight at 4°C. Slides were washed in PBS, stained with secondary antibodies at room temperature for 1 hour, and then incubated with 4′, 6-diamidino-2-phenylindole (Thermo Fisher Scientific, Wilmington, DE) for 2 minutes at room temperature, before mounting with PermaFluor mounting medium (Thermo Fisher Scientific). After staining, images were captured on an LSM 710 confocal microscope (Carl Zeiss, Oberkochen, Germany). Primary antibodies were rabbit anti-Lysozyme (clone EPR2994(2), Abcam, Cambridge, UK), rabbit anti-Chromogranin A (Abcam), or rat anti-CD44 (clone IM7, BD Biosciences, Franklin Lakes, NJ). Secondary antibodies were Alexa Fluor 488 donkey anti-rat IgG or Alexa Fluor 546 donkey anti-rabbit IgG (Thermo Fisher Scientific).

### RNA Isolation and Quantitative PCR

Total RNA was extracted from tissues, organoids, and cultured cells using an RNeasy minikit (Qiagen, Venlo, Netherlands), and cDNA was synthesized using Superscript Ⅱ reverse transcriptase and oligo (dT) primer (Invitrogen, Camarillo, CA). cDNA was amplified with PCR primers (Table 1). Quantitative PCR (qPCR) was performed using PowerUp SYBR Green Master Mix on an Applied Biosystems 7500 real-time PCR system (Thermo Fisher Scientific). The mRNA expression levels were displayed as the expression units of each target gene relative to the expression units of β-actin or GAPDH.

**Table 1:** List of Specific Primer Sets Used for qPCR

Gene symbol	Forward sequence (5′-3′)	Reverse sequence (5′-3′)
Mouse		
GCG	CACGCCCTTCAAGACACAG	GTCCTCATGCGCTTCTGTC
CCK	ACTGCTAGCGCGATACATCC	CCCACTACGATGGGTATTCG
FXR	GAAAATCCAATTCAGATTAGTCTTCAC	CCGCGTGTTCTGTTAGCAT
VDR	CACCTGGCTGATCTTGTCAGT	CTGGTCATCAGAGGTGAGGTC
TGR5	CAGGAGGCCATAAACTTCCA	GTCAGCTCCCTGTTCTTTGC
FGF15	GAGGACCAAAACGAACGAAATT	CAGTCCATTTCCTCCCTGAA
Shp	TCTGCAGGTCGTCCGACTATTC	AGGCAGTGGCTGTGAGATGC
Ibabp	GGTCTTCCAGGAGACGTGAT	ACATTCTTTGCCAATGGTGA
Ostα	ATGCATCTGGGTGAACAGAA	GAGTAGGGAGGTGAGCAAGC
Ostβ	TGACAAGCATGTTCCTCCTG	TGCAGGTCTTCTGGTGTTTCT
Asbt	GACTCGGGAACGATTGTGAT	GGTTCAATGATCCAGGCACT
CYP7a1	AACAACCTGCCAGTACTAGATAGC	GTGTAGAGTGAAGTCCTCCTTAGC
CYP8b1	CTGGCTTCCTGAGCTTATTC	CCCAGTAGGGAGTAGACAAA
β-actin	TGGAATCCTGTGGCATCCATGAAAC	TAAAACGCAGCTCAGTAACAGTCCG
Human		
LKB1	AACGGCCTGGACACCTTCT	CCCTTCCCGATGTTCTCAA
FGF19	CCGACGGCAAGATGCA	TCCTCCTCGAAAGCACAGTCT
Shp	CCCCAAGGAATATGCCTGCC	TAGGGCGAAAGAAGAGGTCCC
GAPDH	GAAGGTGAAGGTCGGAGT	CATGGGTGGAATCATATTGGAA

qPCR, Quantitative polymerase chain reaction.

### RNA-seq Analysis

A library of 1 μg of each isolated total RNA sample was prepared using the TruSeq mRNA Sample Prep kit (Illumina, San Diego, CA). PolyA-selected RNA extraction, RNA fragmentation, random hexamer-primed reverse transcription, and 100-nt paired-end sequencing were performed using the HiSeq4000 platform (Illumina). The libraries were quantified using qPCR under the qPCR Quantification Protocol Guide (KAPA Library Quantification kits) and qualified using the 2100 Bioanalyzer (Agilent Technologies, Santa Clara, CA). For RNA-seq experiments, data processing and statistical analyses were conducted by Macrogen Inc (Seoul, Korea) as previously described.^63^ GSEA was performed using GSEA ver. 4.1.0.^64^^,^^65^ to assess the expression of published signature gene sets for intestinal epithelial lineages.^66^^,^^67^ Data are accessible at the National Center for Biotechnology Information (BioProject ID PRJNA744286).

### Isolating IEC and Crypts

Whole IECs for the aldefluor assay were isolated as described previously.^68^ Briefly, the SI was opened and rinsed with cold PBS. Tissues were incubated in 30 mM of EDTA/PBS on ice for 20 minutes, followed by incubation in fresh 30 mM EDTA/PBS at 37°C for 8 minutes. After the tissue was shaken by hand for 10 minutes, it was removed, and the cells were pelleted at 1,000× g for 5 minutes. Cells were dissociated into single cells by incubation in TrypLE Express (Thermo Fisher Scientific) for 10 minutes at 37°C, before passing through a 40-μm cell strainer. Cells were pelleted and used for the aldefluor assay. To isolate crypts for organoid culture, the SI was opened longitudinally and washed several times with PBS before dissection into 1-cm lengths. Tissues were incubated in 1 mM EDTA/PBS on ice with gentle shaking for 30 minutes followed by incubation with 5 mM EDTA on ice for 1 hour after discarding the solution. Tissues were then transferred to PBS and vigorously shaken with a vortex for 2 minutes before passing through a 100-μm cell strainer. Isolated crypts were pelleted by centrifugation at 250 × g for 3 minutes. Organoid culture was performed as previously reported.^69^

### BA Analysis

Fecal BA excretion was determined by collecting and drying feces from individually housed mice for 48 hours. BA in the liver, SI, LI, cecum, and feces were extracted with 75% ethanol at 50°C for 2 hours. Gallbladders were homogenized in 0.5 mL of PBS. BA was then measured using a total BA kit (Crystal Chem, Downers Grove, IL). BA composition was analyzed by homogenizing 10 mg of freeze-dried SI tissue or feces with 100 μl of 50% methanol (MeOH). Lipid levels of BA were determined using a LC-MS/MS system equipped with an 1290 HPLC (Agilent, Santa Clara, CA) and QTRAP 5500 (AB Sciex, Framingham, MA). Sample preparation and analysis were performed as described previously.^70^

### Cell Culture, Transfection, and Luciferase Assay

Caco-2 (KCLB NO: 30037.1) and HT-29 (KCLB NO: 30038) cells were purchased from the Korean Cell Line Bank (Seoul, Korea). Caco-2 cells were grown in complete Dulbecco’s Modified Eagle Medium supplemented with 10% fetal bovine serum and 1% Antibiotic-Antimycotic (Thermo Fisher Scientific). HT-29 cells were maintained in complete RPMI. For siRNA-mediated gene knockdown, cells were transfected with 25 nM predesigned LKB1 siRNA (SASI_Hs01_00092687, Sigma Aldrich) or negative control siRNA (SN-1011, Bioneer, Daejeon, Korea) using a Lipofectamine RNAiMAX (Thermo Fisher Scientific). The medium was replaced with a fresh complete medium after overnight incubation. The supernatants and cells were harvested at 48 hours post-transfection. For the luciferase assay, 100 ng FXRE-luciferase reporter plasmid (kindly provided by Dr Bart Staels [Institute Pasteur de Lille, Lille, France]) was introduced into Caco-2 cells, followed by LKB1 siRNA transfection overnight. At 24 hours post-transfection, 5 μM GW4064 (Sigma Aldrich) was treated for an additional 24 hours. Luciferase activity was measured using the Luciferase assay system in a Glomax 96 Microplate Luminometer (both from Promega Corporation, Madison, WI).

### Enzyme Immunoassay and Enzyme-linked Immunoabsorbant Assay (ELISA)

Serum CCK level was assessed using a CCK enzyme immunoassay kit (Sigma Aldrich) and FGF19 protein concentration was measured in cell culture supernatants by ELISA (FGF19 Quantikine ELISA kit; R&D Systems, Minneapolis, MN), according to the manufacturer’s protocol.

### Metagenomics

DNA was extracted from the mouse cecum and SI content using QIAamp DNA stool mini kits (Qiagen). Bacterial 16S rDNA sequencing and data analysis was performed at Chunlab Inc as described previously.^63^ All sequences are publicly available at the National Center for Biotechnology Information (BioProject ID PRJNA744286).

### BSH Enzyme Activity Assay

Whole SI content was collected with 5 mL PBS containing 0.05% L-cysteine HCl (Sigma Aldrich). Ten μL SI content was used to impregnate a sterile paper disc (Thermo Fisher Scientific), and placed on MRS agar plates supplemented with 0.05% L-cysteine HCl, 0.5% (w/v) sodium taurodeoxycholic acid, and 0.37 g/L CaCl_2_ (Sigma Aldrich). Plates were incubated under anaerobic conditions at 37°C for 72 hours, and the diameter of the precipitation zones was measured.

### Aldefluor Assay

According to the manufacturer’s instructions, the aldehyde dehydrogenases activity of IECs was determined using the Aldefluor kit (STEMCELL Technologies, Vancouver, BC, Canada).

### Quantification of Retinol and RA

Retinoids in the liver and SI were measured using LC/MS-MS analysis as previously described with minor modifications.^71^ Briefly, 50- to 100-mg tissues were homogenized with 5× volume of PBS on ice, then 20 μL of internal standards (10 μM all-trans-retinol-d_5_ and 4 μM all-trans-RA-d_5_ [both from Toronto Research Chemicals, Toronto, ON, Canada]), were added and mixed well. Retinols and RAs were extracted from the sample solutions through liquid–liquid extraction. Retinoids were analyzed by an LC-MS/MS (1290 UHPLC [Agilent] and QTRAP 5500 mass spectrometer [AB Sciex]), and then separated with solvent A (H_2_O containing 0.1% formic acid) and solvent B (acetonitrile containing 0.1% formic acid). The flow rate was 400 μL/min, and the column temperature was maintained at 23°C. The LC gradient was as follows: for retinol and RA analysis, the initial 70% of B held to 3 minutes, 70% to 78% of B for 9 minutes, 78% to 100% of B for 0.5 minutes, and held at 100% of B for 6.5 minutes before returning to initial conditions. Multiple reaction monitoring was performed in the positive ion mode, and the extracted ion chromatogram corresponding to the specific transition for each retinoid was used for quantification. The calibration range for each retinoid was 1 to 10,0000 nM (r^2^ ≥ 0.99). Data analysis was performed with Analyst 1.7.

### Western Blot

Cultured cell pellets and SI tissues were lysed in RIPA buffer with 1% Halt protease and phosphatase inhibitor cocktail (Thermo Fisher Scientific). The assay was performed in line with a previously described protocol.^72^ Primary antibodies were LKB1 (Santa Cruz Biotechnology, Dallas, TX), FGF19, phospho-AMPKα, or β-actin (all from Cell Signaling Technology, Danvers, MA).

### Glucose Tolerance Test and Insulin Tolerance Test

For the gluten tolerance test, mice were fasted for 16 hours, and injected intraperitoneally with 2 g/kg of glucose. For the insulin tolerance test, after 6 hours of fasting, mice were injected intraperitoneally with 0.75 U/kg of insulin (Sigma Aldrich). Blood samples were taken from the tail at 0, 15, 30, 60, 90, and 120 minutes after injection, and glucose concentration was assessed using a glucometer.

### Comprehensive Laboratory Animal Monitoring System

Mice fed on a HFD for 6 to 7 weeks were placed individually in Comprehensive Laboratory Animal Monitoring System (Columbus Instruments, Columbus, OH) cages and monitored for 4 days. All measurements for each parameter: volume of oxygen consumed (mL/kg/h) and carbon dioxide produced (mL/kg/h), respiratory exchange ratio, and heat (kcal/h) were recorded every 10 seconds.

## Statistics

GraphPad Prism software (GraphPad, La Jolla, CA) was used for statistical analyses. Significant differences between the 2 groups were analyzed with a 2-tailed unpaired *t* test. Multiple groups were analyzed by 1-way and 2-way analysis of variance followed by the Tukey post-hoc test (∗*P* < .05; ∗∗*P* < .01).
